# Understanding the variability of handheld spectral-domain optical coherence tomography measurements in supine infants

**DOI:** 10.1371/journal.pone.0225960

**Published:** 2019-12-11

**Authors:** Kira L. Wang, Xi Chen, Sandra Stinnett, Vincent Tai, Katrina P. Winter, Du Tran-Viet, Cynthia A. Toth

**Affiliations:** 1 Department of Ophthalmology, Duke University, Durham, North Carolina, United States of America; 2 Department of Ophthalmology, Division of Vitreoretinal Diseases and Surgery, Duke University, Durham, North Carolina, United States of America; 3 Department of Biostatistics and Bioinformatics, Duke University, Durham, North Carolina, United States of America; 4 Department of Biomedical Engineering, Duke University, Durham, North Carolina, United States of America; Massachusetts Eye & Ear Infirmary, Harvard Medical School, UNITED STATES

## Abstract

**Purpose:**

Central foveal thickness (CFT) measurements from optical coherence tomography (OCT) scans provide a precise measure of severity of pathologic changes in the fovea, progress of disease and response to treatment. Although these measures are additionally valuable to assess foveal development in infants, their reproducibility is not known. The goal of this retrospective study is to evaluate the variation and reproducibility of CFT measurements using handheld spectral-domain OCT (hh-SDOCT) in supine infants compared to conventional adult tabletop imaging.

**Methods:**

Imaging sessions with multiple macular, volume scans in one eye were selected for analysis from two participant groups: Group 1, 25 imaging sessions from 21 preterm infants without macular edema imaged supine in the nursery using hh-SDOCT (Leica/Bioptigen Envisu C2300, RTP, NC); Group 2, 25 imaging sessions from 25 adults imaged using tabletop Bioptigen SDOCT. For each imaging session, three macular OCT volumes with acceptable image quality were selected for analysis. CFTs were measured using a customized script for automatic segmentation. An expert grader and a typical grader corrected the segmentation lines for the central foveal frame. Coefficient of variations (CV) and intraclass correlation coefficients (ICC) were calculated for graders and systems and compared to the previous literature on OCT reproducibility.

**Results:**

CFT measurements were repeatable and reproducible for both handheld and tabletop SDOCT systems. For handheld, grader ICC (CI) and mean CV were 0.94 (0.90–0.97) and 3.8 (typical) and 0.98 (0.96–0.99) and 2.9 (expert), and for tabletop were 0.91(0.83–0.96) and 2.1 (typical) and 0.92 (0.86–0.96) and 1.9 (expert). Intergrader reproducibility of handheld and tabletop SDOCT systems were ICC(CI) 0.97 (0.95–0.98) and 0.93 (0.89–0.96) respectively, and both are comparable to previously reported reproducibility of tabletop systems.

**Conclusion:**

Handheld SDOCT is a reproducible instrument to measure foveal thicknesses in supine infants. It can be used in clinical research to evaluate foveal changes during retinal development and pathological conditions.

## Introduction

Spectral-domain optical coherence tomography (SDOCT) is a noninvasive imaging system used to evaluate the microanatomical features of the retinas of both pediatric and adult eyes. Tabletop SDOCT is a reproducible system used to image upright, cooperating adults and older children [1–4]. An OCT system has been adapted to handheld use to image supine infants or children who cannot cooperate for tabletop imaging [5]. Although infants provide additional challenges due to their lack of cooperative fixation on a target, the portability and non-invasive nature of handheld SDOCT (hh-SDOCT) makes it a useful tool to image infants in the nursery.

Central foveal thickness (CFT) is a quantitative value measured on OCT that is critical in evaluation of macular edema or retinal layer thinning that may relate to visual prognosis and the development of the cerebral cortex [6, 7]. Relevant to CFT measurements from OCT, infant eyes have several important distinctions from adult eyes, such as an increasing axial length during development, a shallower foveal pit, thinner retina at the fovea, and changing foveal thickness with age [8]. The repeatability and reproducibility of CFT measurements using hh-SDOCT on supine, non-sedated infants is not known. The goal of this study was to evaluate retrospectively the variation and reproducibility of supine hh-SDOCT to measure CFT in infants in comparison to conventional tabletop imaging in fixating adults. This aim was achieved in this study.

## Materials and methods

Imaging sessions with at least three macular, zero-degree (horizontal relative to the upright eye) volume scans in one eye were selected for analysis from two groups of participants: Group 1 included 21 preterm infants from a prior prospective research study of infant retinal OCT imaging without macular edema, retinoschisis or retinal detachment, and who at the time of ROP examinations had been imaged while supine in the nursery using hh-SDOCT (Envisu C2300 Leica/Bioptigen, RTP, NC). The imaging was conducted without sedation and with or without pharmacological dilation. We did not use a lid speculum, but rather, held the eyelid open with the fingertips during retinal imaging. With the lids held open for a shorter period of time than with lid speculum, artificial tears were rarely used. Group 2 included 25 adults from the control group of a prior prospective research study in macular degeneration imaged using tabletop SDOCT (Bioptigen, RTP, NC) [1]. The Duke University Health System institutional review board approved the protocol and the study adheres to the Health Insurance Portability and Accountability Act and all tenets of the Declaration of Helsinki. Written consents were obtained from the adult participants and from the parents or legal guardians of infant participants.

**Fig 1:**
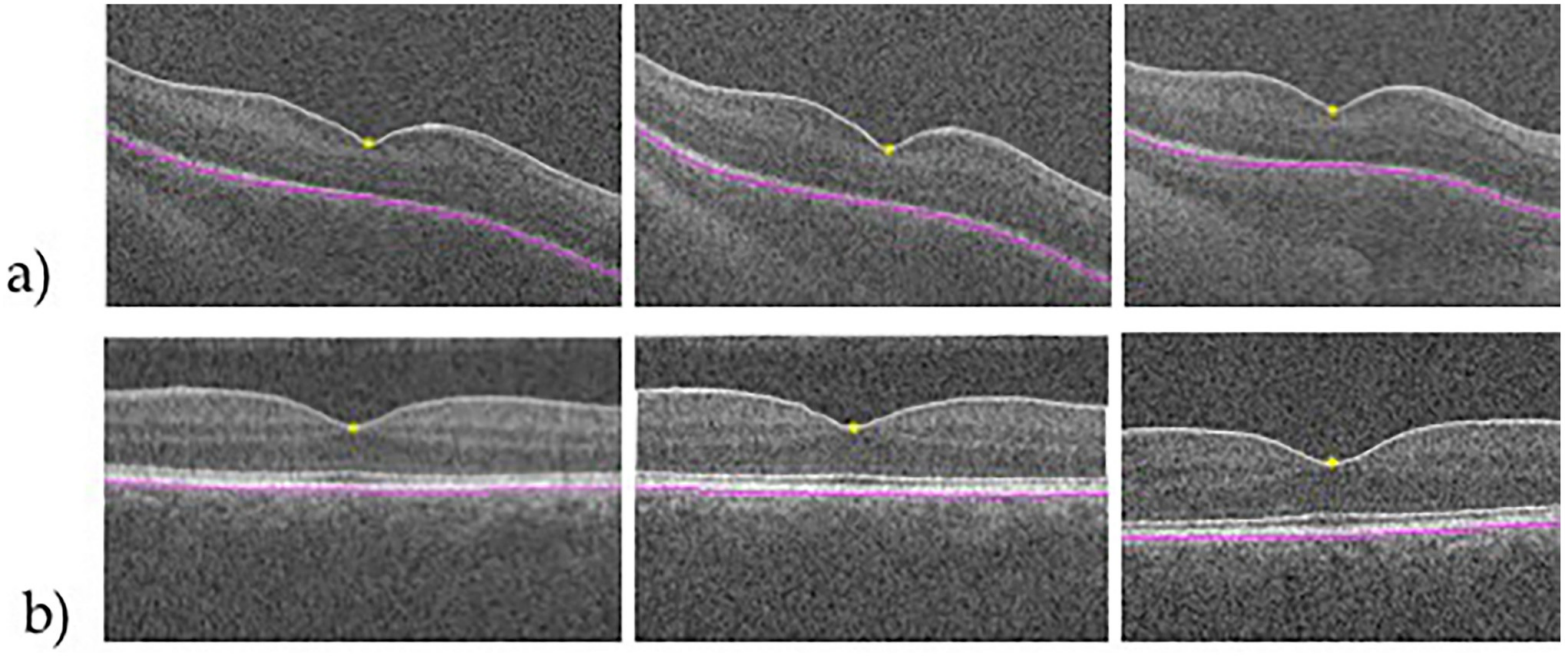
Sample foveal OCT B-scans from infant and adult participant scan volumes. The inner border of internal limiting membrane (white line) and outer border of Bruch’s membrane (purple line) were marked at the foveal center (yellow star) of foveal b-scans from three volumes for each participant: a) a preterm infant imaged using hh-SDOCT (Group 1) and b) a healthy adult imaged using tabletop SDOCT (Group 2). CFT measurements were calculated as the distance between the lines at the foveal center.

Three volumes from each of 25 imaging sessions per group (in four infants, images were from two separate imaging sessions at different eyes/ages) were selected for this retrospective analysis. In the infant group (Group 1), all hh-SDOCT scans were set at 10 x 10 mm for an adult retina which was equivalent to approximately 6.7 x 6.7 mm field-of-view (35–39 weeks), 6.3 x 6.3 mm field-of-view (30–35 weeks), 7.0 x 7.0 mm (39–41 weeks), and 7.3 x 7.3 mm (0–1 month) in the shorter infant eye [9]. In the adult group, the three tabletop SDOCT scans were of different sizes: one 5 x 5 mm, one 8 x 8 mm, and one 10 x 10 mm volume (Fig 1). Foveal frames were selected and CFT measurements were performed separately by an expert (KPW) and a typical (KLW) grader (typical grader is defined as a person who is certified but does not have extensive experience in OCT grading). Foveal frames were chosen based on visual identification of the deepest foveal pit; scan quality was prioritized over tilt of scan. CFT was defined as the distance between the inner border of the internal limiting membrane and the outer border of Bruch’s membrane. For all 150 scans, CFTs were measured using a customized script for automatic segmentation with manual correction by the two graders using Duke OCT Retinal Analysis Program (DOCTRAP) in MATLAB (The MathWorks Inc, Natick, MA) (S1 Dataset).

The coefficient of variations (CV) were calculated over all 25 imaging sessions for both graders and for both handheld and tabletop systems. Several different measures of reproducibility were computed using the intraclass correlation. Separately, for the tabletop expert gradings and for the handheld expert gradings, the intraclass correlation coefficients were computed on the 3 replicates (scans) using the one-way ANOVA random effects model, with 75 total scans each. For the comparison of expert grading with typical grading for both handheld and table-top scans, the patient identifier was given a suffix representing the scan number so that individual scans for the same patient could be matched between graders. Thus, each of the three scans was treated as an independent measurement. Then the intraclass correlation coefficients were computed on the 2 replicates (typical and expert graders) using the one-way ANOVA random effects model, with 150 total scans each comparison.

## Results

Demographic information of infant and adult participants is shown in Table 1. Group 1 included 21 preterm infants (mean age at imaging 39.2 ± 3.6 weeks) while Group 2 included 25 adults (mean age at imaging 65.5 ± 5.6 years). As expected because of foveal development, infants exhibited a greater range in CFT measurements than did mature adults (Table 1).

**Table 1:** Demographic information of infant (group 1) and adult (group 2) participants.

Variable	Group 1 (n = 21)	Group 2 (n = 25)
**Age**	(weeks)	(years)
Mean (SD)	39.2 (3.6)	65.5 (5.6)
Range (Min., Max)	(34, 45)	(54, 77)
**Gender, no. (%)**		
Male	9 (43)	13 (52)
Female	12 (57)	12 (48)
**Race, no. (%)**		
White	5 (24)	23 (92)
Black	13 (62)	0
Asian	1 (5)	2 (8)
Multiracial	2 (9)	0
**Ethnicity, no. (%)**		
Not Hispanic or Latino	20 (95)	25 (100)
Hispanic or Latino	1 (5)	0
**CFT (μm)**		
Mean (SD)		
Typical Grader	157.1 (35.6)	244.5 (18.4)
Expert Grader	155.2 (36.2)	246.2 (19.4)
Range (Min., Max.)		
Typical Grader	(96.0, 225.6)	(201.1, 277.9)
Expert Grader	(91.2, 220.8)	(201.1, 296.2)

SD = standard deviation, CFT = central foveal thickness

**Fig 2:**
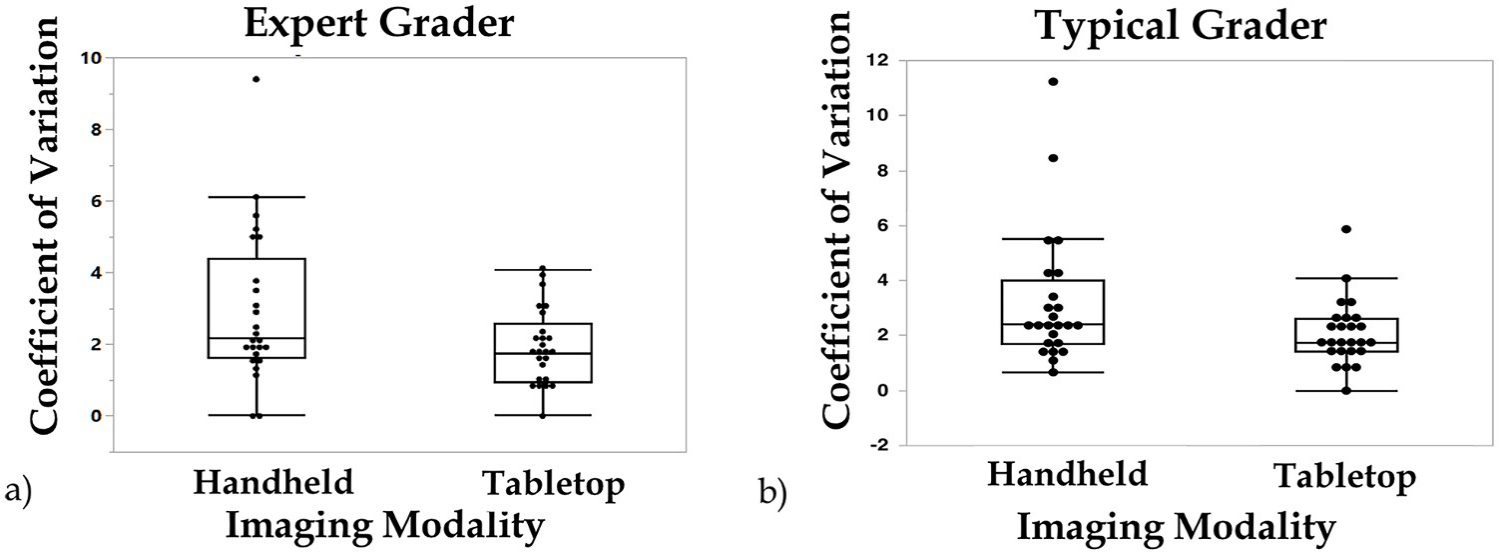
**Box and whisker plots of coefficients of variation for** a) expert grader measurements from handheld images versus tabletop images, and b) typical grader measurements from handheld images versus tabletop images (one CV outlier (19.6) is not shown for typical grader of handheld images for proper sizing of the box and whisker plot).

For the typical grader, the ICC (95% CI) was 0.94 (0.90, 0.97) for hh-SDOCT (Group 1) and 0.91 (0.83, 0.96) for tabletop SDOCT (Group 2). The mean CV was 3.8 for Group 1 and 2.1 for Group 2. For the expert grader, the ICC (95% CI) was 0.98 (0.96, 0.99) for Group 1 and 0.92 (0.86, 0.96) for Group 2. The mean CV was 2.9 for Group 1 and 1.9 for Group 2. A comparison of the ICCs and CVs of Groups 1 and 2 indicates that handheld and tabletop systems are both reproducible, while hh-SDOCT exhibited wider variation around the median coefficient of variation than tabletop SDOCT (Fig 2).

**Fig 3:**
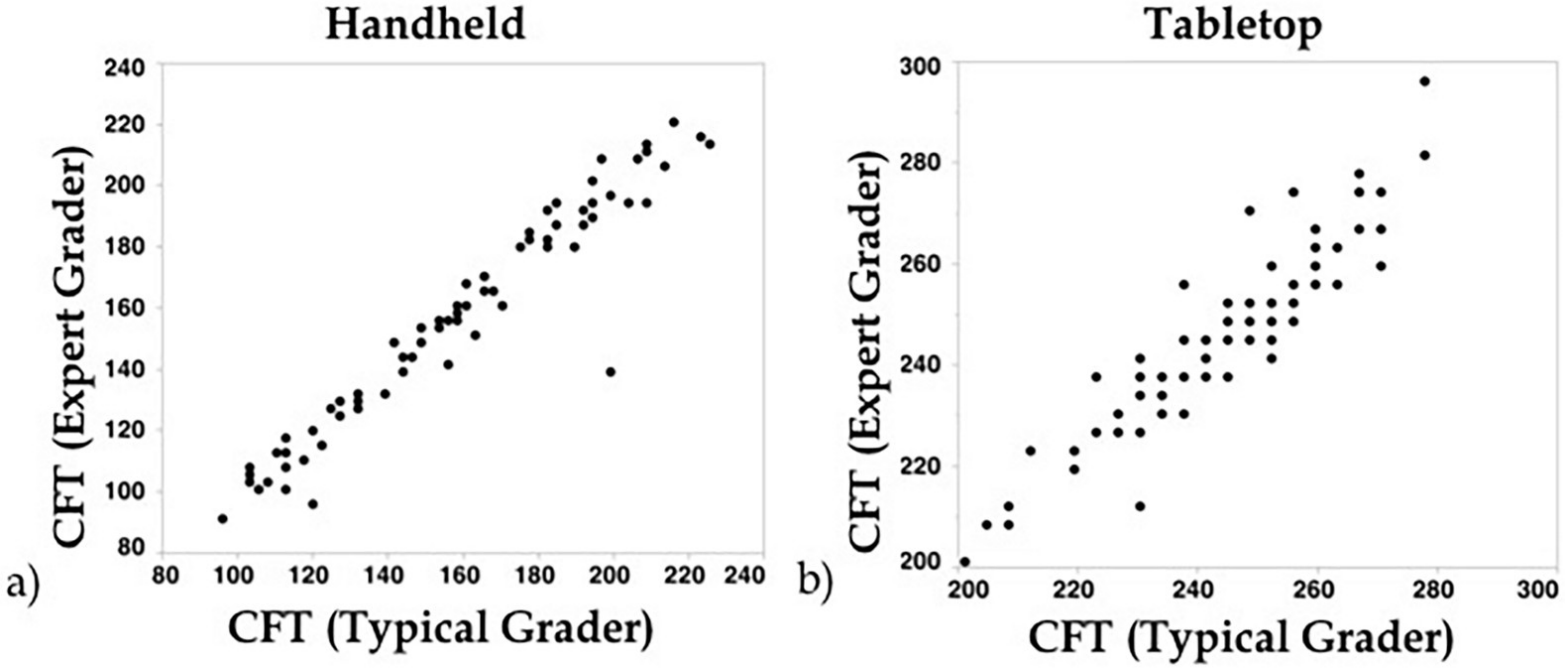
**Comparison of central foveal thickness measurements** between expert and typical graders for a) hh-SDOCT in preterm infants and b) tabletop SDOCT in adults. Note a greater range in foveal thicknesses in infants (~80–240 μm) than adults (~200–300 μm) was observed.

Between expert and typical graders, there was excellent agreement on CFT measurements for both handheld and tabletop SDOCT scans. The intergrader reproducibility for Group 1 (hh-SDOCT) was ICC = 0.97 (95% CI, 0.95, 0.98) and for Group 2 (tabletop) was 0.93 (0.89, 0.96). The mean absolute difference in CFT measurements was 1.9 ± 9.2 μm for Group 1 and 1.7 ± 6.9 μm for Group 2. The expert and typical foveal thickness measurements for each group are shown in Fig 3.

## Discussion

Prior studies have shown that tabletop SDOCT systems are reproducible and reliable tools for assessing retinal thicknesses. The reproducibility in these studies was described by ICC values ranging from 0.92 to 0.99 for Topcon, Cirrus, and RTVue systems [2–4]. Handheld-SDOCT is an adaptation of the tabletop system that is useful for imaging non-fixating infants and children in a supine position [10]. While macular images captured by hh-SDOCT are of comparable quality to those produced by tabletop SDOCT [10], reproducibility of handheld OCT systems have not been reported prior to this study. One might hypothesize that hh-SDOCT measurements in infants would be more variable, due to the lack of fixation and lack of stabilization of the participant’s head in a chin rest. In our study, CFT measurements of both handheld and tabletop SDOCT systems were reproducible, while the handheld SDOCT system yielded wider variations. Despite this, the variation and reproducibility of our CFT measurements are similar to the values for other tabletop SDOCT systems previously reported in the literature [2–4, 9]. These findings imply that the handheld SDOCT system is a reproducible imaging modality that could generate reliable CFT measurements in infants and children.

Many factors can lead to variations in retinal thickness measurements on OCT imaging. These include the chosen platform such as handheld or tabletop system, the segmentation algorithm, and anatomical differences (axial length and macular curvature) between individual patients [9]. OCT image quality may also play a significant role (hence only scans with acceptable or better image quality were included). In this study, both platform and patient characteristics differed between the groups. For handheld system imaging in infants in a nursery, unique challenges such as manual alignment, examiner variability, probe tilt, hand movement, infant eye movement, and image rotation may also cause variations in these images [11]. Thus, it was notable how reproducible CFT measurements were despite these variables.

It is important for clinicians and researchers to recognize that infant foveal thickness changes during development. Thus, for example we identified a greater range in CFT measurements in infants than in adults (Fig 3). On SDOCT images, preterm infants exhibit a shallower fovea, thinner inner retinal layers such as the inner nuclear and inner plexiform layers, and lack of photoreceptor sublayers, leading to a thinner photoreceptor layer at the fovea [12]. During maturation, the retinal layers of the infant eye become thicker and the fovea deepens [12]. The greater range of CFT measurements in infants compared to adults may be the cause of a higher ICC value observed in hh-SDOCT compared to the tabletop system. Despite these distinctions in the developing infant eye, hh-SDOCT appears to be a reproducible measure that can be useful in the nursery to evaluate retinal development and diseases. We recognize that this study is limited by its retrospective nature and it does not evaluate the same participants across systems. A prospective reproducibility study comparing adult CFTs across tabletop and handheld systems is currently underway.

In addition to uncooperative children, other causes of fixation loss, such as extensive macular pathology, eye movement abnormalities, or poor vision in both eyes, may affect scan quality. Indeed, prior studies in infants and children with nystagmus showed that handheld OCT could yield reliable macular measurements.[13] Our study in infants to some extent mimic older individuals with fixation losses and indicates that handheld SDOCT could also be potent tool to assess CFT in these patients.

In summary, both handheld and tabletop SDOCT systems reproducibly assess CFT measurements in infants and adults. To our knowledge, this is the first study to evaluate the reproducibility of hh-SDOCT in comparison to tabletop SDOCT. Handheld SDOCT appears to be a reproducible system to assess CFT and could potentially be used as an endpoint in clinical studies.

## Supporting information

S1 Dataset The handheld OCT variability data—Grouped file tabulates the complete data from the imaging sessions for group 1 (infants) and group 2 (adults) with a Data Dictionary. (XLSX)

## References

[1] Farsiu S, Chiu SJ, O’Connell RV, Folgar FA, Yuan E, Izatt JA, et al Quantitative classification of eyes with and without intermediate age-related macular degeneration using optical coherence tomography. Ophthalmology. 2014;121(1):162–72. Epub 2013/08/29. 10.1016/j.ophtha.2013.07.013 . 23993787 PMC3901571

[2] Leung CK, Cheung CY, Weinreb RN, Lee G, Lin D, Pang CP, et al Comparison of macular thickness measurements between time domain and spectral domain optical coherence tomography. Invest Ophthalmol Vis Sci. 2008;49(11):4893–7. Epub 2008/04/30. 10.1167/iovs.07-1326 . 18450592

[3] Menke MN, Dabov S, Knecht P, Sturm V. Reproducibility of retinal thickness measurements in healthy subjects using spectralis optical coherence tomography. Am J Ophthalmol. 2009;147(3):467–72. Epub 2008/11/20. 10.1016/j.ajo.2008.09.005 . 19026403

[4] Ho J, Sull AC, Vuong LN, Chen Y, Liu J, Fujimoto JG, et al Assessment of artifacts and reproducibility across spectral- and time-domain optical coherence tomography devices. Ophthalmology. 2009;116(10):1960–70. Epub 2009/07/09. 10.1016/j.ophtha.2009.03.034 . 19592109 PMC2757525

[5] Vinekar A, Sivakumar M, Shetty R, Mahendradas P, Krishnan N, Mallipatna A, et al A novel technique using spectral-domain optical coherence tomography (Spectralis, SD-OCT+HRA) to image supine non-anaesthetized infants: utility demonstrated in aggressive posterior retinopathy of prematurity. Eye (Lond). 2010;24(2):379–82. Epub 2010/01/08. 10.1038/eye.2009.313 . 20057510

[6] Rossi EA, Roorda A. The relationship between visual resolution and cone spacing in the human fovea. Nat Neurosci. 2010;13(2):156–7. Epub 2009/12/20. 10.1038/nn.2465 . 20023654 PMC2822659

[7] Lee H, Purohit R, Patel A, Papageorgiou E, Sheth V, Maconachie G, et al In Vivo Foveal Development Using Optical Coherence Tomography. Invest Ophthalmol Vis Sci. 2015;56(8):4537–45. 10.1167/iovs.15-16542 . 26200492

[8] Cook A, White S, Batterbury M, Clark D. Ocular growth and refractive error development in premature infants with or without retinopathy of prematurity. Invest Ophthalmol Vis Sci. 2008;49(12):5199–207. 10.1167/iovs.06-0114 . 19036998

[9] Maldonado RS, Izatt JA, Sarin N, Wallace DK, Freedman S, Cotten CM, et al Optimizing hand-held spectral domain optical coherence tomography imaging for neonates, infants, and children. Invest Ophthalmol Vis Sci. 2010;51(5):2678–85. Epub 2010/01/13. 10.1167/iovs.09-4403 . 20071674 PMC2868489

[10] Scott AW, Farsiu S, Enyedi LB, Wallace DK, Toth CA. Imaging the infant retina with a hand-held spectral-domain optical coherence tomography device. Am J Ophthalmol. 2009;147(2):364–73.e2. Epub 2008/10/09. 10.1016/j.ajo.2008.08.010 . 18848317

[11] Dayani PN, Maldonado R, Farsiu S, Toth CA. Intraoperative use of handheld spectral domain optical coherence tomography imaging in macular surgery. Retina. 2009;29(10):1457–68. 10.1097/IAE.0b013e3181b266bc . 19823107 PMC3515871

[12] Vinekar A, Mangalesh S, Jayadev C, Maldonado RS, Bauer N, Toth CA. Retinal Imaging of Infants on Spectral Domain Optical Coherence Tomography. Biomed Res Int. 2015;2015:782420 Epub 2015/07/06. 10.1155/2015/782420 . 26221606 PMC4506845

[13] Lee H, Proudlock F, Gottlob I. Is Handheld Optical Coherence Tomography Reliable in Infants and Young Children With and Without Nystagmus? Invest Ophth Vis Sci. 2013;54(13):8152–9. 10.1167/iovs.13-13230 24222299 PMC4054919

